# qPCR in gastrointestinal stromal tumors: Evaluation of reference genes and expression analysis of *KIT *and the alternative receptor tyrosine kinases *FLT3*, *CSF1-R*, *PDGFRB*, *MET *and *AXL*

**DOI:** 10.1186/1471-2199-11-100

**Published:** 2010-12-20

**Authors:** Jana Fassunke, Marie-Christine Blum, Hans-Ulrich Schildhaus, Marc Zapatka, Benedikt Brors, Helen Künstlinger, Reinhard Büttner, Eva Wardelmann, Sabine Merkelbach-Bruse

**Affiliations:** 1 Department of Pathology, University of Bonn Medical Center, Bonn, Germany; 2 Division of Molecular Genetics, DKFZ, Heidelberg, Germany; 3 Department of Integrative Bioinformatics and Systems Biology, DKFZ, Heidelberg, Germany; 4 Department of Hematology and Oncology, Otto-von-Guericke-University Magdeburg, Germany

## Abstract

**Background:**

Gastrointestinal stromal tumors (GIST) represent the most common mesenchymal tumors of the gastrointestinal tract. About 85% carry an activating mutation in the *KIT *or *PDGFRA *gene. Approximately 10% of GIST are so-called wild type GIST (wt-GIST) without mutations in the hot spots. In the present study we evaluated appropriate reference genes for the expression analysis of formalin-fixed, paraffin-embedded and fresh frozen samples from gastrointestinal stromal tumors. We evaluated the gene expression of *KIT *as well as of the alternative receptor tyrosine kinase genes *FLT3*, *CSF1-R*, *PDGFRB*, *AXL *and *MET *by qPCR. wt-GIST were compared to samples with mutations in *KIT *exon 9 and 11 and *PDGFRA *exon 18 in order to evaluate whether overexpression of these alternative RTK might contribute to the pathogenesis of wt-GIST.

**Results:**

Gene expression variability of the pooled cDNA samples is much lower than the single reverse transcription cDNA synthesis. By combining the lowest variability values of fixed and fresh tissue, the genes *POLR2A*, *PPIA*, *RPLPO *and *TFRC *were chosen for further analysis of the GIST samples. Overexpression of *KIT *compared to the corresponding normal tissue was detected in each GIST subgroup except in GIST with *PDGFRA *exon 18 mutation. Comparing our sample groups, no significant differences in the gene expression levels of *FLT3*, *CSF1R *and *AXL *were determined. An exception was the sample group with *KIT *exon 9 mutation. A significantly reduced expression of *CSF1R*, *FLT3 *and *PDGFRB *compared to the normal tissue was detected. GIST with mutations in *KIT *exon 9 and 11 and in *PDGFRA *exon 18 showed a significant *PDGFRB *downregulation.

**Conclusions:**

As the variability of expression levels for the reference genes is very high comparing fresh frozen and formalin-fixed tissue there is a strong need for validation in each tissue type. None of the alternative receptor tyrosine kinases analyzed is associated with the pathogenesis of wild-type or mutated GIST. It remains to be clarified whether an autocrine or paracrine mechanism by overexpression of receptor tyrosine kinase ligands is responsible for the tumorigenesis of wt-GIST.

## Background

Gastrointestinal stromal tumors (GIST) are the most common mesenchymal tumors of the gastrointestinal tract and are characterized by the expression of the KIT receptor (stem cell factor receptor, CD117) and to a lesser extent of PDGFRA (platelet derived growth factor receptor alpha), representing two closely related receptor tyrosine kinases (RTK) [1,2]. The majority of GIST shows oncogenic mutations either in *KIT *or *PDGFRA *[3,4]. Mainly, mutations in exon 9 or 11 of the *KIT *gene or in exon 18 of *PDGFRA *lead to ligand independent, constitutive activation of the kinase function [5]. About 60% of all GIST carry an exon 11 mutation of *KIT *which encodes the juxtamembrane domain of the receptor possessing an autoinhibitory function [6,7]. Less common mutations in *PDGFRA *(~ 10%) are detected in GIST that often display gastric location and epithelioid morphology [2].

In a minority of cases (10-15%) no mutations in the known *KIT *or *PDGFRA *hot spots are detected although these tumors express the KIT protein. This subgroup is called wild type GIST (wt-GIST) and comprises tumors in pediatric patients, in patients affected by the Carney triad, neurofibromatosis type 1 (NF1) associated GIST and a subset of sporadic adult GIST [8-11]. The pathogenetic mechanisms underlying wt-GIST are poorly understood and there is limited benefit of imatinib therapy in these patients [12]. Therefore the identification of additional genetic factors contributing to the pathogenesis of GIST may help to find new concepts of individualized therapy.

Recently, the *BRAF *mutation p.V600E was found in 4-13% of wt-GIST [13-15]. For another subgroup of wt-GIST including pediatric tumors, a strong IGF1R expression combined partly with gene amplification was described [16-18]. Two other alternative RTK probably involved in the pathogenesis of GIST are AXL and MET. Both kinases have been shown to be upregulated in GIST resistant to treatment [19]. AXL is a member of the Ufo/AXL subfamily and activates the same signaling pathway as KIT. The tyrosine kinase domain of *MET *is mutated in sporadic papillary renal carcinomas. Some mutations in the *MET *gene are located in codons homologous to those in *KIT *and it is suggested that these missense mutations lead to constitutive activation of the MET protein [20]. To develop additional therapy approaches it would be of interest to know whether this RTK also plays a role in wt-GIST.

Besides KIT and PDGFRA, CSF1R (colony stimulating factor 1 receptor), FLT3 (fms like tyrosine kinase 3) and PDGFRB (platelet derived growth factor receptor β) belong to the same family of type III RTK. These five tyrosine kinases show a homologous structure and a comparable function in activation, proliferation and suppressing apoptosis [21-23].

Aberrant expression and mutations in either *CSF1R*, *FLT3 *and *PDGFRB *or their ligands have been described in several malignant diseases. Overexpression of CSF1R is found in epithelial tumors such as breast and ovarian cancer [24]. The translocation t(1;2) of its ligand CSF1 leads to the development of the tenosynovial giant cell tumor [25].

Aberrantly expressed FLT3 is observed at high levels in a spectrum of hematologic malignancies [26]. Additionally, in AML an internal tandem duplication in the transmembrane domain of *FLT3 *was identified which leads to constitutive activation of its kinase domain. It seems that this mutation is not present or very rare in GIST [27].

PDGFRB is overexpressed in malignant peripheral nerve sheath tumors (MPNST) and chordomas [28,29].

To analyse expression profiles of certain tumors for research and diagnostic purposes, qPCR (quantitative PCR) is frequently applied because of its reproducibility and high sensitivity [30,31]. This method is based on the normalization of the target gene expression on stably expressed internal reference genes. A major challenge is the application of suitable reference genes which have to be tested and verified under defined experimental conditions [32,33]. Ideal reference genes have to be non-regulated, stable and not affected by biological or experimental conditions. The target gene is amplified together with the reference gene in order to minimize experimental variability concerning reverse transcription enzymatic efficiencies, PCR efficiency, amount of starting material and differences between human tissues. The reference gene and the target gene should have very robust and stable expression profiles to ensure accurate normalization and interpretation of results. The most stable expressed gene from a set of genes can be identified by geNorm [34], a software program which additionally provides the number of genes required to calculate a robust normalization factor based on the geometric mean of these genes.

Typical reference genes regulate basic and ubiquitous cellular functions and are responsible for the cellular maintenance, e.g. *GAPDH *or ß-actin. However, these commonly used reference genes vary considerably in different tissue types or under different experimental conditions [35,36]. There is no standard reference gene for all kinds of tissue types. To our best knowledge only few studies investigated the alteration of stability of reference genes in different mesenchymal tumour entities.

In the present study we attempted to identify suitable reference genes in gastrointestinal stromal tumors by using a set of sixteen reference genes which are currently applied in qPCR procedures. Furthermore we evaluated the gene expression of *KIT *as well as the alternative RTK *FLT3*, *CSF1-R*, *PDGFRB*, *AXL *and *MET *in mutated and non-mutated gastrointestinal stromal tumors by qPCR using the identified reference genes. The study was focussed on wt-GIST compared to samples with mutations in *KIT *exon 9 and 11 and *PDGFRA *exon 18 in order to evaluate whether overexpression of these alternative RTK might contribute to the pathogenesis of wt-GIST.

## Methods

### Samples

A total of 107 samples were included into this study. All specimens were obtained in the years 2005 and 2006 under approved ethical protocols and with informed consent from each patient. All samples were fixed in neutral-buffered formalin prior to paraffin embedding. 20 samples from normal tissue (i.e., muscularis propria of stomach and gut) as control group and 87 GIST representing different mutational subgroups were evalutated. 20 samples of wt-GIST, 7 samples of wt-GIST associated with neurofibromatosis type 1, 20 samples with exon 9 mutation in *KIT*, 20 samples with exon 11 mutation in *KIT *and 20 samples with exon 18 mutation in *PDGFRA*). Sequence analysis of *KIT *(exons 8, 9, 11, 13, 14, 15 and 17) and *PDGFRA *(exons 12, 14 and 18) was carried out as described earlier [1,37,38]. Additionally, in all samples the wild type status of the *BRAF *gene was ascertained. All GIST samples were stained immunohistochemically for CD117. Four normal tissues from the gastrointestinal region were available as fresh frozen and formalin-fixed, paraffin-embedded samples.

### RNA extraction and cDNA synthesis

Prior to RNA extraction, paraffin-embedded tissues were cut into 10 μm sections and mounted on glass-slides. Six slides of each specimen were used for RNA extraction. The sections were deparaffinized by extracting twice in xylene for 10 min at room temperature. Rehydration was done in 100% ethanol, 90% ethanol, 80% ethanol and 70% ethanol made with DEPC-water for 10 min each. Tumor areas previously marked on a H&E slide were scraped from the sections with a sterile scalpel. Sections were transferred into a sterile 1.5 ml tube. Extraction and purification of RNA was done using the RNeasy FFPE KIT (Qiagen, Hilden, Germany) according to the manufacturer's recommendations. After tumor localization by H&E staining fresh frozen material was rasped into 10 μm thick pieces and RNA purification was carried out using the RNeasy Kit (Qiagen, Hilden, Germany). Both RNeasy Kits contain a step of DNase treatment. Finally RNA from fresh and from fixed tissue was eluted in water. The quantification was done spectrophotometrically (NanoDrop, PeqLab Technology, Erlangen, Germany). 500 ng of RNA from each sample was reverse-transcribed using a random-hexamer primer and Avian Myoblastosis Virus reverse transcriptase (AMV-RT) according to the manufacturer's protocol (Qiagen). cDNA from those four fresh frozen and corresponding formalin-fixed, paraffin-embedded (FFPE) control samples was generated in only one reverse transcription and loaded on the reference low density arrays (see below). Further cDNA samples were generated from the four FFPE control tissues, pooled within one patient and loaded onto a second independent reference low density array.

### Identification of reference genes

The reference genes used here were preselected because of their constitutive, non-regulated stable expression over a wide spectrum of tissues. But nevertheless the preselected reference genes are not suitable for every kind of tissue and therefore need to be analyzed prior to use in a certain study. The detection of suitable reference genes was carried out using TaqMan Low Density Arrays (TLDAs, Microfluidic Cards, Applied Biosystems, Darmstadt, Germany). These arrays are prefabricated 384-well cards where gene-specific primer and probe sets are spotted in small reaction chambers during manufacturing. The cards have 8 separate loading ports leading into 48 wells each. In this study, arrays with triplicates of 16 putative reference genes were used, so 8 different samples could be analyzed. The list of assays is given in table 1. For each sample, 500 ng cDNA was mixed with 2× TaqMan Universal PCR Master Mix (Applied Biosystems). 100 μl of this mixture was loaded into each port and distributed into the reaction chambers by centrifugation. The card was sealed and the quantitative PCR (qPCR) was performed on an ABI PRISM HT 7900 (Applied Biosystems) sequence detection system. After pre-incubation for 2 min at 50°C and 10 min at 95°C, the PCR reaction was performed (15 s at 94°C followed by 60 s at 60°C, 40 cycles). The fluorescent signal was measured in each cycle.

**Table 1:** Selected candidate reference genes for gene expression analysis.

Abbreviation	Gene name	Cellular function	
18S rRNA	18S ribosomal RNA	ribosome subunit	X03205.1
ACTB	ß-actin	cytoskeleton	NM_001101.3
B2M	ß-2-microglobulin	major histocompatibility complex	NM_004048.2
GAPDH	glyceraldehyde-3-phosphate dehydrogenase	glycolysis enzyme	NM_002046.3
GUSB	ß-glucuronidase	glycosaminoglycan degradation	NM_000181.2
HMBS	hydroxymethylbilane synthase	heme production	NM_000190.3
HPRT1	hypoxanthine ribosyltransferase	metabolic salvage of purines	NM_000194.2
IPO8	importin 8	intracellular protein transport	NM_006390.2
PGK1	phosphoglycerate kinase 1	glycolysis enzyme	NM_000291
POLR2A	DNA-dependent RNA polymerase II	transcription	NM_000937.3
PPIA	peptidyl-prolyl isomerase A	protein folding	NM_021130
RPLPO	ribosomal phosphoprotein, large, P0	ribosome	NM_001002
TBP	TATA-box-binding protein	transcription factor	NM_003194
TFRC	transferrin receptor	cellular iron uptake	NM_021009.4
UBC	ubiquitin C	posttranslational modification	NM_003406.3
YWHAZ	tyrosine 3-/tryptophan 5-Monooxygenase-activation protein, zeta isoform	signal transduction	NM_003404

### Analyses of gene expression by qPCR

qPCR analysis was performed using the assays-on-demand products (Applied Biosystems) listed in table 2. These gene-specific qPCR assays consist of a pair of unlabeled PCR primers and a FAM labeled specific probe. According to the manufacturer of these assays, probe and primer sets that would amplify pseudogenes are excluded in the process of development. Reactions were carried out in a reaction volume containing 5 μl PCR Master Mix (Applied Biosystems), 0.5 μl forward and reverse primer mix, 500 ng cDNA ad 10 μl A.dest. Triplicate reactions were carried out for each transcript. Control reactions were performed using a minus RT preparation and a sample with A.dest instead of RNA. PCR conditions were the same as for the TaqMan Low Density Arrays.

**Table 2:** Gene-specific qPCR assays.

Gene symbol	Name	Chr	Function	Assay-on-demand
CSF1R	colony stimulating factor 1 receptor	5	tyrosine protein kinase receptor	Hs00234617_m1
FLT3	fms-related tyrosine kinase 3	13	tyrosine protein kinase receptor	Hs00174690_m1
KIT	v-kit Hardy-Zuckerman 4 feline sarcoma viral oncogene homolog	4	tyrosine protein kinase receptor	Hs00174029_m1
PDGFRß	platelet-derived growth factor receptor, beta	5	tyrosine protein kinase receptor	Hs00387362_m1
MET	met proto-oncogene	7	hepatocyte growth factor receptor	Hs00179845_m1
AXL	AXL receptor tyrosine kinase	19	tyrosine protein kinase receptor	Hs00242357_m1

### Statistical analyses

The geNorm applet for Microsoft Excel was used to determine the most stable genes among the sixteen candidate reference genes. Raw Cq values were converted into relative quantities for analysis with geNorm, where the highest relative quantity for each gene is set to 1. The program selects from a panel of candidate reference genes the two most stable genes or a combination of multiple stable genes for normalization. The gene expression stability (M) value is based on the combined estimate of intra- and intergroup expression variations of the genes studied and takes the PCR efficiency into account. The limited M-value is 1.5. The most stable genes are stepwise selected from the investigated gene panel to estimate how many reference genes should be used. The normalization factors define the optimal number of reference genes required for a precised normalization design. The analysis of the expression data of tumor samples compared to control samples was performed with the REST software (Relative Expression Software Tool) [39]. The software normalizes the measured Cq-values of the target genes with those of the reference genes and compares the expression data of tumor and control samples by considering the PCR efficiency and the mean crossing point deviation.

## Results

### RNA quality

RNA quality of the samples was inspected on a 1% agarose gel. The concentration and purity of the RNA was characterized by the mean A_260/280 _ratio and was on average 1.99 for fresh frozen as well as for formalin fixed, paraffin-embedded samples and reflected pure and protein-free RNA.

### Expression variability of cDNA synthesis

**Figure 1:**
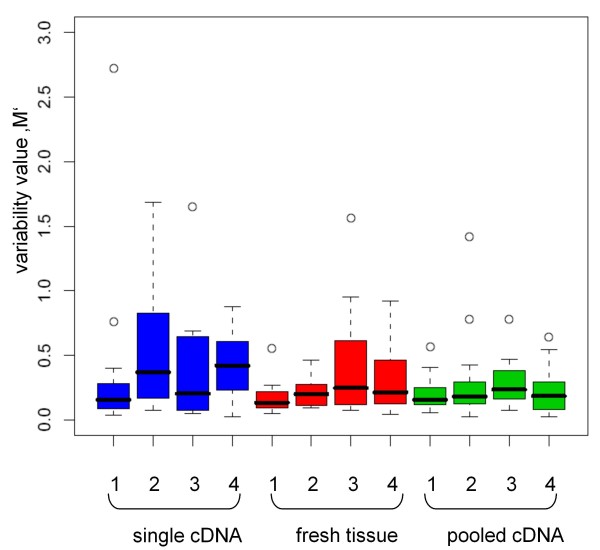
**Variability of technical replicates**. Expression variability of cDNA from a single reverse transcription synthesis (blue), cDNA from pooled reverse transcription synthesis (green) and fresh tissue cDNA (red) from a single reverse transcription synthesis. The whiskers are representing the range of the data, the median is shown as a black line within a bar. Circles are representing the outliers.

Four normal control FFPE tissue samples from the gastric and bowel wall were used to determine suitable reference genes. One TLDA was done with cDNA from only one reverse transcription for each sample. As it is assumed that cDNA synthesis from FFPE tissue varies strongly in efficiency, pooled cDNA synthesis samples from the same FFPE tissue were used for a second TLDA. The pooled cDNA preparation showed an essentially lower variance in contrast to the cDNA samples from only one reverse transcription (Figure 1). The median of the M-values ranges from 0.1 to 0.2, thus the variability of the technical replicates is low. Within a single specimen the range is also very small. The median of the cDNA samples from a single reverse transcription ranges from 0.1 to 0.4. However, each cDNA sample from one reverse non-pooled transcription showed a high variance of the values (median of M-values 0.1 to 0.8, Figure 1). Therefore the variance of the samples can be lowered by cDNA pooling. The median of the cDNA samples from one reverse transcription from fresh frozen tissue ranged from 0.1 to 0.2 and can be compared to the median of the pooled cDNA from the FFPE tissue. Within a single specimen the range is higher than in the pooled cDNA samples, but smaller than in the cDNA from only one reverse transcription from the FFPE samples.

### Identification of suitable reference genes for normalization

**Figure 2:**
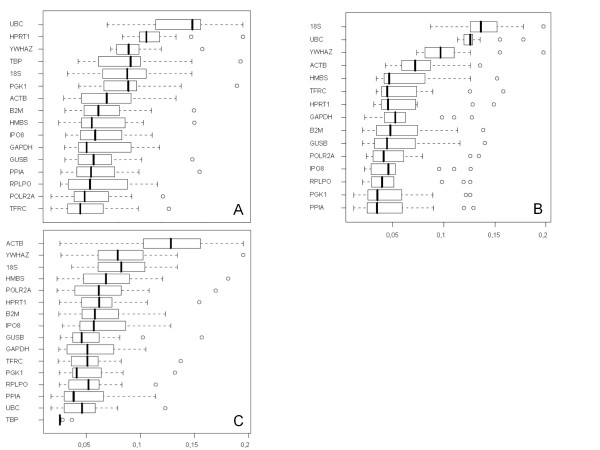
**Expression variability of reference genes**. Analysis of reference gene expression on TaqMan Low Density Arrays. a) cDNA from a single RT-transcription. b) pooled cDNA samples from normal tissue. The genes *POLR2A*, *TFRC*, *PPIA *and *RPLPO *had the lowest expression variability. c) cDNA from fresh frozen tissue. *RPLPO*, *PPIA *and *UBC *had the lowest expression variability, *TBP *was not expressed in the tissue. On the x-axis, the stability values are plotted. Whiskers represent range of data of 4 samples.

**Figure 3:**
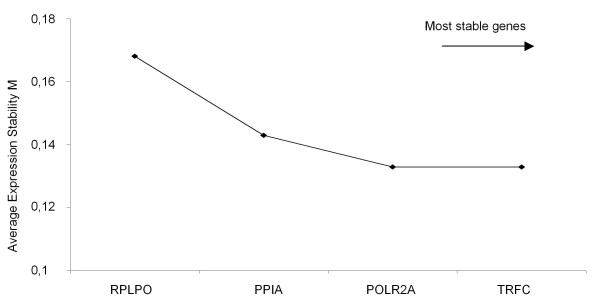
**GeNorm analysis of selected reference genes**. Calculation of the average expression stability of the remaining 4 candidate reference genes for normalization in GIST by geNorm analysis. The least stable gene with the highest 'M'-value is indicated from left to right on the x-axis. After combining the data of the single RT-transcription cDNA and the pooled cDNA the stepwise exclusion of the least stable reference genes resulted in POLR2A and TRFC.

All 16 putative reference genes had a high expression stability and the 'M'-value (0.02-0.06) was clearly below the 'M'-cutoff-value of 1.5. By stepwise exclusion of genes, the expression stability value 'M' is calculated (data not shown) and the expression stability rises with the exclusion of further genes. Samples generated from only one reverse transcription, showed *POLR2A*, *TFRC*, *RPLPO *and *GAPDH *as the most stable genes (Figure 2a). The average stability value was between 0.05 and 0.07. The range of the values was 0.01-0.15. The most stable genes for pooled cDNA samples were *PGK1*, *PPIA*, *RPLPO *and *IPO8 *with a median of 0.04-0.05 (Figure 2b). The range of the values was lower and varied between 0.01 and 0.09. All in all the gene expression variability of the pooled cDNA samples is much lower compared to the single reverse transcription cDNA synthesis. The gene *UBC *is the most stable one in fresh frozen tissue with the smallest value range (0.01-0.8), but *RPLPO *and *PPIA *belong also to the most stable genes in fresh frozen tissue with a median of 0.03-0.045 and the range of the values was 0.1-0.12 (Figure 2c). TBP was not expressed in the tissue. By combining the lowest variability values of fixed and fresh tissue, the genes *POLR2A*, *PPIA*, *RPLPO *and *TFRC *were detected (Figure 3). Hence these four genes were used for further analysis with the GIST samples.

### Expression profiles of KIT, FLT3, CSF1-R, PDGFRB, AXL and MET

Six patient groups (NT = normal tissue -control, WT = wild type, Ex9 = *KIT *Exon 9 mutated, Ex11 = *KIT *Exon 11 mutated, NF1 = wild type and neurofibromatosis type 1, Ex18 = *PDGFRA *Exon 18 mutated) were used to analyze the gene expression of *KIT*, *FLT3*, *CSF1-R*, *PDGFRB*, *AXL *and *MET*. Here, the REST analysis with *POLR2A *is shown exemplarily. The REST analysis of the target genes *KIT, CSF1R*, *FLT3*, *PDGFRB, AXL *and *MET *with the reference genes *RPLPO*, *PPIA *and *TRFC *showed the same significant results for the differential expression or at least the same trend in mutated and non-mutated GIST (data not shown). As suspected, we found a significant overexpression of *KIT *in exon 9 and exon 11 mutated GIST in comparison with normal tissue. Also a significantly lowered expression of *PDGFRB *in both groups compared to normal tissue was shown. The same effect was observed in *PDGFRA *exon 18 mutated GIST compared to normal tissue (table 3).

**Table 3:** Expression analysis of target genes.

	Gene	Factor	p-value		Gene	Factor	p-value
**NT↔WT**	KIT	5.9+	0.095	**NT↔NF1**	KIT	17+	**0.003**
	CSF1R	3.99-	0.217		CSF1R	2.99-	0.294
	FLT3	2.43-	0.498		FLT3	3.985-	0.179
	PDGFRB	5.79-	0.087		PDGFRB	2.827-	0.196
	AXL	2.1-	0.66		AXL	2.5+	0.53
	MET	8.7-	**0.034**		MET	6.5-	0.13
							
**NT↔Ex11**				**NT↔Ex18**			
	KIT	11.7+	**0.011**		KIT	1.66+	0.373
	CSF1R	4.65-	0.155		CSF1R	4.7-	0.051
	FLT3	2.383-	0.41		FLT3	2.113-	0.263
	PDGFRB	6.3-	**0.046**		PDGFRB	4.925-	**0.007**
	AXL	3-	0.27		AXL	1.35-	0.58
	MET	17.81-	**0.001**		MET	7.7-	**0.001**
							
**NT↔Ex9**				**Ex11↔WT**			
	KIT	5.261+	**0.05**		KIT	1.981-	0.232
	CSF1R	13.98-	**0.003**		CSF1R	1.164+	0.861
	FLT3	5.38-	**0.022**		FLT3	1.023-	0.971
	PDGFRB	12.78-	**0.001**		PDGFRB	1.087+	0.920
	AXL	3.4-	0.08		AXL	1.4+	0.6
	MET	9.766-	**0.001**		MET	2+	0.33

Expression analysis of *KIT*, *CSF1R*, *FLT3*, *PDGFRB*, *AXL *and *MET *was carried out in six GIST cohorts (NT = normal tissue, WT = wild type, Exon 9, Exon 11, NF1 = neurofibromatosis type 1, Exon 18). Significant variances are in bold. The expression data were calculated using the REST software with *POLR2A *as reference gene. NT: normal tissue; p < 0.05; +/- indicates up- or downregulation

**Figure 4:**
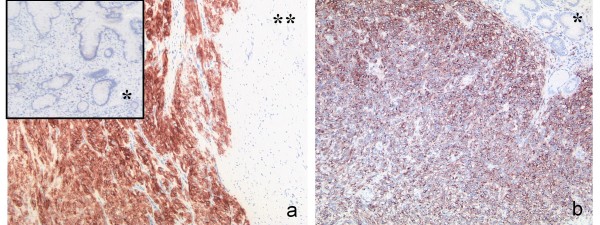
**Immunohistochemical staining for CD117 in wild type and mutant GIST**. a) NF1 associated wild type GIST located in the small intestine with corresponding normal mucosa (*) and muscularis propria (**). b) GIST located in the stomach with a mutation in exon 11 of the *KIT *gene with corresponding normal mucosa (*).

On closer inspection of normal tissue and wt-GIST a clear overexpression of *KIT *in the tissue of wild type tumors was shown. The same effect, but even stronger, could be detected by comparing NF1-associated wt-GIST with normal tissue. Concerning *KIT *expression, the tumors without mutation behave like *KIT *mutated GIST (table 3). This was in accordance with immunohistochemical staining (Figure 4).

The other target genes, *FLT3*, *CSF1R*, *PDGFRB*, *AXL *and *MET *showed lower expression levels in the tumor tissue compared to normal tissue except *AXL *in NF1 associated GIST. *MET *was even significantly reduced in wt-GIST, *KIT *exon 9, 11 and *PDGFRA *exon 18 mutated GIST. No changes in expression levels were detected comparing normal tissue and wt-GIST with *KIT *exon 11 mutated GIST, indicating that both groups possess similar expression levels for the analyzed receptors. None of the groups showed significant expression alterations for *CSF1R *and *FLT3*. An exception is the group of *KIT *exon 9 mutated GIST. A lower expression of *CSF1R*, *FLT3*, *PDGFRB *and *MET *compared to normal tissue was identified in contrast to *KIT*, which showed a significant overexpression. In summary, overexpression of *KIT *was detected in each GIST subgroup compared to the corresponding normal tissue by using the preassigned reference gene *POLR2A *(table 3). GIST with a mutation in exon 18 of *PDGFRA *did not show a significant upregulation of *KIT *compared to normal tissue. GIST with a mutation in *KIT *exon 9, 11 and in the *PDGFRA *exon 18 showed significant *PDGFRB *downregulation.

## Discussion

Most gastrointestinal stromal tumors exhibit mutations in exon 11 of the *KIT *gene. This exon encodes for the juxtamembrane domain of the receptor which possesses an autoinhibitory function. In wt-GIST, activating mutations are found neither in the *KIT *nor in the *PDGFRA *gene. Clinical treatment with the tyrosine kinase inhibitor imatinib targets the ATP binding site in the kinase domain of the KIT receptor. In wt-GIST, response to treatment is often poor [12] although most of them overexpress the KIT protein. A screen for activating mutations in the juxtamembrane domain of the alternative receptor tyrosine kinases CSF1R, FLT3, PDGFRB in 30 wt-GIST samples yielded only wild type sequences (data not shown) in agreement with previously published results [40]. Therefore we investigated in this study whether the expression of alternative receptor tyrosine kinases may contribute to the pathogenesis of wt-GIST and thus may help to identify wt-GIST subgroups with different response to imatinib and elucidate novel therapeutic targets.

The pathogenesis of several malignant tumors is associated with overexpression of CSF1R and PDGFRB [24,28]. Imatinib inhibits cell invasion in malignant peripheral nerve sheath tumors by blocking PDGFRB [41] and it has been found to have antitumor activity in patients with chordoma [42]. In our study, the gene-expression level of *CSF1R*, *FLT3 *and *PDGFRB *was determined in a cohort of 87 GIST samples. Furthermore, we assessed the expression of *AXL *and *MET*, two receptor tyrosine kinases which were found to be alternatively activated in therapy resistant GIST [19].

Gene expression analysis by qPCR requires suitable reference genes. The expression of reference genes like *GAPDH *or *BETA-ACTIN *is regulated differentially depending on the tissue type. Therefore they are not suited as univocal reference genes [35,43]. The determination of reference genes with stable expression in the experimental system used is essential to ensure accurate normalization and interpretation of results.

Whereas fresh tissue is frequently not available for genetic analysis, FFPE material is the standard. After formalin-fixation and paraffin-embedding of tissue, the isolated RNA is often heavily fragmented. In our study we used fresh as well as fixed material from the gastrointestinal tract to determine suitable reference genes and to analyze whether their expression levels are comparable. We then validated the reference genes by qPCR in our GIST cohort.

As reported also by others [44,45] the variability of expression levels for the reference genes was very diverse comparing fresh frozen and formalin-fixed tissue. Therefore, we decided to validate separate reference genes for each tissue type.

The genes *TRFC*, *POLR2A, PPIA *and *RPLPO *were validated as appropriate reference genes for FFPE tissue. For fresh frozen tissue, *PPIA *and *RPLPO *were also found to be suitable. Additionally, *UBC *is suited as a reference gene in fresh frozen tissue. The variability of the reference genes for fresh frozen tissue was lower than for fixed tissue. To overcome the problem of high variability in FFPE tissue, we pooled two independent cDNA syntheses from one sample as recommended in the MIQE guidelines [30,46]. The MIQE guidelines give considerations for a consistent application of the qPCR technology including experimental details, data analysis and reporting principles. Unequal efficiency of cDNA synthesis might be a reason for deviation. Additionally, we tried to select consistent patient material for our cohort by choosing paraffin blocks having the same age. It was shown by Bibikova [45], that Cq-values in qPCR experiments depend on the age and condition of the tissue blocks. Because the expression value depends also on amplicon length [30,44], only primer sets generating amplicons of about 100 bp were chosen for qPCR. This corresponds to the fragment length of degraded RNA between 100 and 200 bp. The application of the MIQE guidelines results in a minimum variability for reference genes. In summary, considering the MIQE guidelines FFPE material can be used reliably for expression analysis in GIST, but the use of separate reference genes for FFPE tissue is indispensable.

Our qPCR analysis included GIST with wild type sequences in the hot-spot regions of *KIT *and *PDGFRA *(wt-GIST), *KIT *exon 9 and exon 11 mutated samples, *PDGFRA *exon 18 mutated samples, NF-1-associated wt-GIST and normal tissue controls. The wt-GIST and the samples with the two different *KIT *mutations showed a significantly increased expression of *KIT *in contrast to the normal tissue. The results correspond to the immunohistochemical stainings of our samples and were in agreement with published data [47]. It was shown immunohistochemically that *PDGFRA *mutated GIST have only a slightly increased protein expression of *KIT *[13]. Our data revealed the same trend on RNA level compared to the normal tissue. Additionally, we could show in all groups of mutated GIST a significantly reduced expression of *PDGFRB *compared to the normal tissue. This could be due to the concomitant increase of *KIT *expression. When comparing NF-1-associated GIST without *KIT *mutation with wt-GIST without NF-1 association the latter showed a lower expression of *KIT*. Thus, the results of the two wt-GIST groups give a heterogenous profile, which suggests that different genomic events may be responsible for the development of these tumors.

Comparing our sample groups with each other, no significant difference in the gene expression levels of *FLT3*, *CSF1R *and AXL were determined. An exception was seen in the sample group with *KIT *exon 9 mutation. Here, a significantly reduced expression of *CSF1R*, *FLT3 *and *PDGFRB *compared to the normal tissue was detected. The results lead us to the assumption that *KIT *exon 9 mutated GIST play a special role compared to GIST carrying other mutations. Interestingly, *KIT *exon 9 mutated GIST need a double daily dose of the tyrosine kinase inhibitor imatinib to be effectively treated [48]. Furthermore, they develop preferentially in the small intestine but only rarely in the stomach where the majority of GIST are detected [49].

All qPCR data were calculated four times with *TRFC*, *POLR2A, PPIA *and *RPLPO *as reference genes using the REST software. Concordant results with the four reference genes are based on the extensive and complex preselection of our cohort and the preparation of cDNA synthesis according to the MIQE guidelines.

## Conclusions

In summary, we conclude that none of the alternative receptor tyrosine kinases analyzed here are associated with the pathogenesis of wild type or mutated GIST. It remains to be clarified whether an increased expression of receptor tyrosine kinase ligands is responsible for tumorigenesis of wt-GIST as it is described for dermatofibrosarcoma protuberans (DFSP) and tenosynovial giant cell tumor (TGCT) [25,50]. Further studies are needed to elucidate the role of ligand-driven pathogenesis in wt-GIST.

## Authors' contributions

MCB, JF and HK carried out the molecular genetic studies and JF wrote and drafted the manuscript. SMB and HUS developed the design of the study. SMB coordinated the study together with EW and HUS. BB and MZ performed the statistical analysis of the expression data. EW and HUS participated in the diagnosis and selection of tumor material. RB revised the manuscript for important intellectual content and approved the final manuscript. All authors read and approved the manuscript.
